# A dataset for material-specific packaging volumes in Finland in 2020

**DOI:** 10.1016/j.dib.2026.113082

**Published:** 2026-07-15

**Authors:** Henna Jylhä, Annika Johansson, Jani Salminen

**Affiliations:** Finnish Environment Institute Syke, Latokartanonkaari 11, 00790 Helsinki, Finland

**Keywords:** Packaging accounts, Fiber packaging, Glass packaging, Metal packaging, Plastic packaging, Wood packaging

## Abstract

The European Packaging and Packaging Waste Regulation sets targets for reducing the use of packaging materials and for improving packaging recycling. Monitoring related to these targets requires comparable data on total packaging volumes in each EU member state. Packaging accounting methodology, which relies on annually reported packaging data obtained from producer responsibility organizations, and standard economic statistics compiled by national statistical office, provides a solid approach to quantifying the total volume of packaging placed on the market within a country. This data article presents an accounting of fiber, glass, metal, plastic, and wood packaging placed on the market in Finland in 2020 and an extrapolation of the data to 2018–2019 and 2021—2024. These highly disaggregated industry and material-specific packaging accounts can be used in further analyses of national packaging flows between industries.

## Graphical abstract

**Figure:**
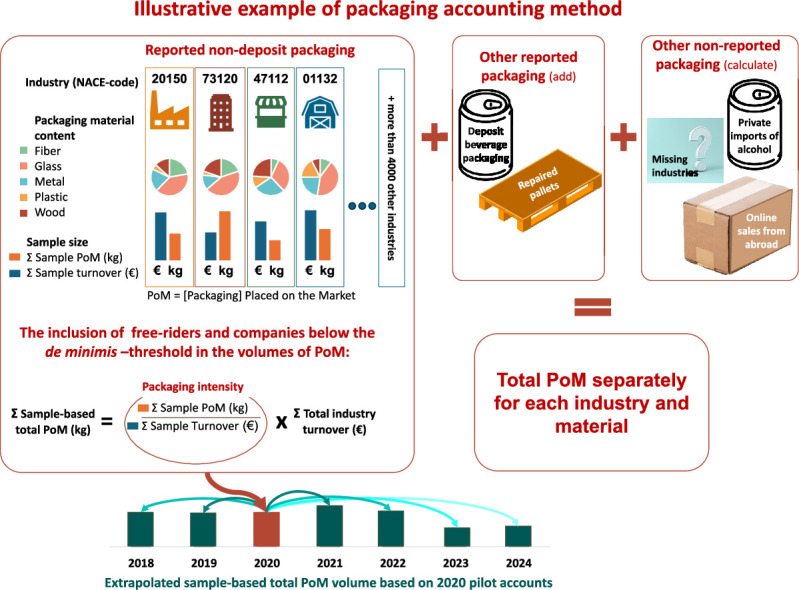

Specifications Table

**Table:** 

Subject	Earth & Environmental Sciences
Specific subject area	Packaging waste, recycling, packaging materials, national packaging accounting, accounting methods
Type of data	Table, Analyzed
Data collection	Reported packaging volumes for 2020 were obtained from producer responsibility organizations. Standard economic statistics by Statistics Finland were used to extrapolate the reported packaging volumes to cover also non-reported packaging volumes. These packaging accounts were completed by adding packaging volume estimates for international online shopping and private imports by using multiple data sources, such as trade statistics and data. The accounts were extrapolated to 2018–2019 and 2021–2024 using packaging intensities and inflation-adjusted turnover statistics compiled from four individual statistics provided by Statistics Finland.
Data source location	Finland, Finnish Environment Institute
Data accessibility	Repository name: Zenodo Data identification number: 10.5281/zenodo.20052357 Direct URL to data: https://doi.org/10.5281/zenodo.20052357
Related research article	H. Jylhä, A. Johansson, J. Sorvari, J. Salminen, A novel method of accounting for plastic packaging waste, Waste Manag., 196 (2025), 42–50. https://doi.org/10.1016/j.wasman.2025.02.022 [1]

## 1 Value of the Data

• The packaging accounts accurately capture the packaging volumes placed on the market (PoM), which are considered equal to the volumes of packaging waste according to calculation rules set in EU legislation.
• The dataset was compiled using a new method developed by Jylhä et al. [1], which combines packaging data reported under Extended Producer Responsibility (EPR) requirements and standard economic statistics produced by Statistics Finland.
• The highly disaggregated industry and material-specific packaging data reveal the origins of packaging flows and can be used for modeling flows between industries and into different waste streams. Here, 418 industries and five packaging materials were considered. The dataset differentiates between consumer and business packaging.
• The extrapolation of the packaging accounts by using the packaging intensity (kg/€ of turnover) and inflation-adjusted turnover statistics of the target provides an important reference for compliance with the requirements of the Packaging and Packaging Waste Regulation (PPWR) with base year 2018 [2]. The extrapolation covered years 2018–2019 and 2021–2024.
• The methodology presented in this data article is applicable in EU countries and beyond. It is a useful approach for reporting packaging PoM and national recycling rates to the European Commission (EC), and it helps the member states meet the obligations set in PPWR.

## 2 Background

EC packaging waste statistics indicate that packaging quantities are increasing, meanwhile insufficient re-use, collection, and recycling of packaging waste create barriers to achieving EU recycling targets [2]. To address this trend, the EU introduced the PPWR, which aims to reduce the negative impacts of packaging and packaging waste on the environment and on human health.

Monitoring the PPWR requires consistent reporting from EU countries. Total volumes of packaging PoM are needed to identify any reductions in packaging waste and the achievement of recycling targets. A lack of transparency and harmonization in data collection methods across the EU hampers comparisons between the member states’ progress toward meeting the PPWR targets and thus undermines their equal treatment [3].

The packaging accounting method in Jylhä et al. [1] enables the total volumes of packaging waste to be calculated in accordance with the PPWR using a PoM approach. It addresses relevant data gaps, including packaging PoM by free riders (companies that avoid producer responsibilities) and by companies whose turnover falls below the *de minimis* threshold and which are therefore exempt from reporting requirements [4]. However, while Jylhä et al. [1] addresses plastic packaging in 2020, similar accounts for all packaging materials and for surrounding years are needed—especially for 2018, which is the base year for the PPWR’s packaging waste reduction target.

## 3 Data Description

The dataset described in this data article is an Excel workbook comprising an Info Sheet; Sheets 1 and 2, which present industry-specific accounts for non-deposit packaging PoM in 2020; Sheet 3, which summarizes the total packaging volume for 2020; Sheet 4, which presents the statistics and indices used for the extrapolation of packaging accounts; and Sheets 2018–2024, which present industry-specific packaging accounts for the years 2018–2024 extrapolated from 2020 data. Packaging volumes are presented by material: fiber, glass, metal, plastic, and wood. Although the plastic packaging accounts for 2020 were previously presented by Jylhä et al. [1], they are included in the sheets here to demonstrate the extrapolation and to improve the usability of the dataset.

**Sheet 1** presents industry-specific accounts for non-deposit fiber, glass, metal, plastic, and wood packaging in 2020 (sample-based packaging volumes). Accounts are compiled separately for consumer and business packaging and for material-specific total packaging volumes. Business packaging accompanies products intended for companies, including sales, transportation, and grouped packaging. Consumer packaging includes the packaging for the consumer products and the packaging filled at the point of sale (such as paper and plastic bags, boxes, and single-use containers). If the same packaging is sold to both consumers and businesses, it is reported as consumer packaging [5]. The packaging volumes in Sheet 1 contain both reported packaging and calculated estimates of packaging PoM by free riders and companies whose turnover is below the *de minimis* threshold. Together, these packaging volumes cover an average of 81% of the total volume of packaging PoM (33–95%, depending on the material), while the remaining 19% is associated with other packaging streams, such as deposit packaging, private imports, and online shopping from abroad. To ensure data confidentiality, the published packaging volumes have been aggregated such that each reporting unit contains at least 12 individual reporting companies. The packaging accounts cover 418 industries, of which 40 are presented individually and 378 are combined into 87 aggregated units.

**Sheet 2** presents estimated packaging accounts for 98 industries without any registered companies in the extended producer responsibility (EPR) customer register in 2020 (i.e., missing industries), but which are presumed to engage in packaging activities and thus to fall within the scope of EPR obligations. Packaging volumes have been calculated individually by material and separately for consumer, business, and total packaging, similar to Sheet 1.

**Sheet 3** presents the total volumes of fiber, glass, metal, plastic, and wood packaging PoM in 2020, separated into three reported and four unreported packaging flows. The reported flows concern non-deposit and deposit packaging, as well as repaired wooden pallets, which are added to the volume of packaging PoM for recycling rate calculations. The non-reported packaging flows include packaging PoM by free-riders and companies below the *de minimis* threshold (two flows), as well as packaging associated with online sales from abroad and private imports of alcohol. The distributions of consumer and business packaging are not specified in the sheet.

**Sheet 4** presents the statistics and specific indices used to adjust the turnover of each industry for inflation.

**Sheets 2018–2024** present the extrapolations of industry-specific packaging accounts for the years 2018–2024; 2020 is included for validation purposes.

## 4 Experimental Design, Materials, and Methods

This data article applies the method used for compiling the plastic packaging accounts for 2020 in Finland introduced by Jylhä et al. [1]. The method relies largely on existing datasets and thus does not require new data collection. The original data, however, were not intended for accounting and research purposes and further data processing and refining has therefore been necessary. In this data article, this method has been applied to calculate both industry-specific and total volumes of packaging materials other than plastic (fiber, glass, metal, and wood), which are also covered by the EPR scheme and reporting obligations. Three novel elements are introduced in this data article; first, for packaging of imported alcohol, the calculations are completed using data on glass and metal beverage packaging and on group packaging for cans and wine bottles. Second, for wooden packaging, the volume of repaired palettes reported by manufacturers is added to the palettes PoM. Third, a packaging data extrapolation method is described and the resulting extrapolated packaging accounts for 2018–2019 and 2021—2024 are presented.

### 4.1 Accounts for non-deposit packaging

The method for non-deposit packaging is based on packaging data reported to Finnish Packaging Recycling RINKI Ltd—a non-profit service company that is jointly owned by Finnish industries and retail trades—which, among other tasks, collects packaging data on behalf of producer responsibility organizations according to EPR obligations [6]; these concern all companies that pack products in Finland or import packed products for the Finnish market. Until 2024, companies with turnovers of less than €1 million (the *de minimis* threshold) were exempted from the EPR obligations.

Reported packaging volumes form a sample of all the packaging PoM, and they are accompanied by information about the industry according to NACE classification and turnover of the reporting companies. NACE is the system for statistical classification of economic activities in the European community, in which each industry is given a NACE code; the system contains hierarchy according to which industries can be aggregated or disaggregated [7]. The method assumes that a given type of packaging is used in a similar manner within each industry. First, industry-specific sample representativeness (*SR_i_*) are calculated using Eq. (1):

(1) $$SR_{i}=\frac{T_{s,i}}{T_{i}}$$

where *T_s,i_* is the industry-specific sum of the turnovers of the companies forming the sample and *T_i_* is the total turnover of an industry.

Next, the industry-specific total packaging volume PoM (*V_i_*), including the packaging of free riders and companies below the *de minimis* threshold (upscaling), is calculated according to Eq. (2):

(2) $$V_{i}=\frac{V_{s,i}}{SR_{i}}$$

where V_s_*_,i_* is the industry- and material specific packaging volume in the sample. The method does not differentiate between free riders and companies below the threshold. If the industry’s packaging activity is deemed occasional (non-representative), no upscaling is applied. These industries were identified by using packaging material purchasing data [8] as described by Jylhä et al. [1] and two criteria: first, low representativeness in terms of turnover (<10% representativeness in the sample) and second, the low number of companies forming the sample (no more than two).

**Fig. 1:**
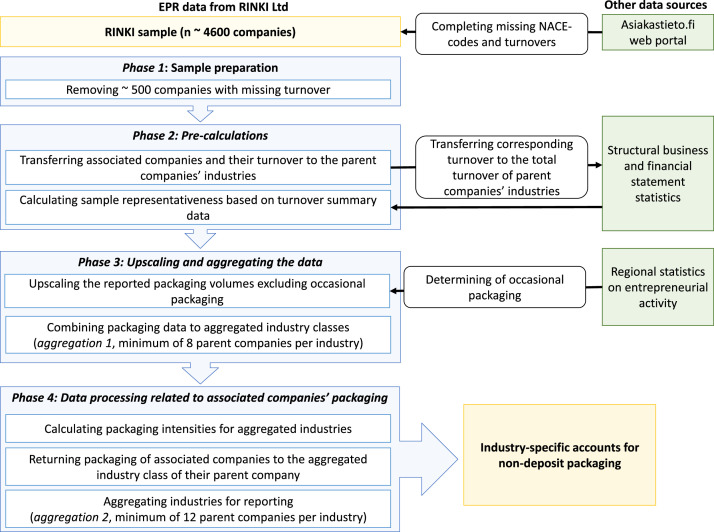
Process of compiling industry-specific packaging accounts for non-deposit plastic packaging according to Jylhä et al. [1] (sample-based packaging PoM). The method is also applicable to other packaging materials.

The two major challenges related to the methodology are 1) that not all companies report within their own industries because their parent companies may submit a joint report on behalf of one or more associated companies, even when the parent company’s industry affiliation differs from that of its associates, and 2) to protect data confidentiality, some industries must be combined, entailing aggregation into groups of at least eight reporting companies. For reporting, further aggregation into groups of at least 12 reporting companies is required to maintain company-level data confidentiality. The phases of compiling industry-specific packaging accounts for non-deposit packaging are presented in Fig. 1, and the results are in Sheet 1 of the dataset.

The method does not consider industries that have no companies represented in the EPR scheme. However, based on packaging material purchase data [8], 98 industries are deemed to have placed packaging on the market despite not being affiliated with the EPR scheme. For each of these industries, an expert assessment was conducted to identify a matching industry with similar packaging usage from the industries listed in Sheet 1 to allow an estimate of the volume of packaging PoM by industry. The estimate was generated using the value of purchased packaging material [8] and industry turnover data [9] as described in detail by Jylhä et al. [1] (see Sheet 2 of the dataset for the results).

### 4.2 Packaging imported through online shopping and private imports

To estimate packaging volumes associated with households’ online shopping from abroad, the consumer packaging intensities for retail sale via mail order houses or the internet (NACE code 47910; kg of packaging material per the total turnover of the industry of €1.07 billion [9]) are multiplied by the estimated turnover of consumer products purchased from abroad (€2.04 billion) in 2020 according to the Finnish Commerce Federation [1]. It is assumed that the packaging types for online shopping from abroad are similar to those of domestic online shopping. The results are presented on Sheet 3 of the dataset.

An estimate of packaging volume accompanying private imports of alcohol is based on the volumes of different alcoholic beverages (beer, ciders, other mild alcohol drinks, wines, mixed drinks, spirits) privately imported into Finland [10], partially confidential data regarding alcohol packaging distribution in Finland [11,12], and average weights of different packaging types [11,12,13]. In the absence of reliable data for other imported products, the related packaging volumes are excluded from the analysis.

Packaging material *P* associated with each privately imported beverage packaging type *j* (*P_j_*) is calculated by using Eq. (3):

(3) $$\sum P_{j}=\sum \frac{V_{\text{total},i{\;}^{*}}x_{j}}{V_{\text{packaging},j}\;}*M_{\text{packaging},j}$$

where *V*_total,_*_i_* is the total volume of each imported beverage *i* (see Table 1), *x_j_* is the share of different packaging types for each beverage (see Table 2), *V*_packaging,_ *_j_* is the volume of different packaging types (see Table 2), and *M*_packaging,_ *_j_* is the average weight of different packaging types (see Table 3).

**Table 1:** The total volumes of different alcoholic beverages privately imported to Finland in 2020 [10].

Beverage	*V*_total,_*_i_* (million liters)
Beer	13.3
ciders and other mild alcohol drinks	7.7
mild wines	4.8
fortified wines and mixed drinks	1.0
spirits	2.6

**Table 2:** The shares and volumes of the different packaging types j and group packaging typed k used for different beverages. The data obtained from Palpa [11]* is partially confidential and therefore not shown.

Beverage packaging type *j*	*x_j_* (%)	*V*_packaging,_*_j_* (L)	Data source
beer, cider and other mild alcohol drinks, in 0.33 L plastic bottles	data not shown*	0.33	[11]
beer, cider and other mild alcohol drinks, in 0.33 L metal cans	data not shown*	0.33	[11]
beer, cider and other mild alcohol drinks, in 0.33 L glass bottles	data not shown*	0.33	[11]
mild wines, in 0.75 L glass bottles	40	0.75	[12]
mild wines, in 3-liter bag-in-box containers	60	3	[12]
fortified wines and mixed drinks in 0.75 L glass bottles	100	0.75	[12]
spirits, in 0.5 l glass bottles	data not shown*	0.5	[11]
spirits, in 0.5 l plastic bottles	data not shown*	0.5	[11]

Group packaging type k	xk, group packaging (%)	Vgroup packaging, k (L)	Data source

metal cans in group packaging	100	24×0.33 L	[12]
mild wine bottles in group packaging	50	12×0.75 L

**Table 3:** Average weights of different beverage packaging and group packaging types.

Beverage packaging material	Beverage packaging type *j*	*M*_packaging,_*_j_* (g)	Data source
Plastic	bottle (0.33 L)	23.9	[11]
Plastic	bottle (0.5 L)	36.1	[11]
Plastic	bag-in-box container (3 L) - interior	40.0	[12]
Fiber	bag-in-box containers (3 L) - exterior	120.0	[12]
Glass	bottle (0,75 L)	470.0	[11]
Glass	bottle (0.5 L)	470.0	[11]
Glass	bottle (0.33 L)	340.0	[11]
Metal	can (0.33 L)	13.0	[11]

Group packaging material	Group packaging type *k*	*M*_group packaging,_*_k_* (g)	Data source

Fiber	sparkling wine box 12×0,75 L bottles	325.0	[13]
Fiber	box for 24×0.33 L cans	150.0	[12]

All the metal cans (0.33 L) and half of the glass wine bottles (0.75 L) are associated with group packaging. Packaging material *P* associated with group packaging *k* (*P_k_*) is calculated by using Eq. (4):

(4) $$\sum P_{k}=\sum \frac{V_{\text{total},i}*x_{j}*x_{k}}{V_{\text{group}\;\text{packaging},k}}*M_{\text{group}\;\text{packaging},k}$$

where *x_k_* is the share of different packaging types in different group packaging (see Table 2), *V*_group packaging,_ *_k_* is the total volume of cans or bottles in the group packaging (see Table 2), and *M*_group packaging,_ *_k_* is the mass of the group packaging (see Table 3).

The results are presented on Sheet 3 of the dataset.

### 4.3 Extrapolation of the packaging accounts

Sample-based packaging accounts from 2020 are extrapolated to 2018–2019 and 2021–2024 by multiplying the packaging intensities (kg of packaging / € of turnover) by inflation-adjusted turnover statistics. The packaging volumes of aggregated industries are computationally disaggregated for the calculations, and each industry is then extrapolated individually. Disaggregation is based on the relative shares of turnover in each aggregate. After extrapolation, the industries are re-aggregated into the original groupings.

The original turnover statistics for 2018–2024 were retrieved from structural business and financial statement statistics [9]. The non-specialized wholesale (NACE 46901) and retail trade (NACE 47111, 47113, and 47301) industries have been merged because wholesale companies report on behalf of retail trade companies in the majority of cases, as detailed in Jylhä et al. [1].

Three different statistics from Statistics Finland have been used for the inflation adjustments of turnover statistics: The producer price index (basic price index for domestic supply measures) [14], the producer price indices for services (services for all end users) [15], and the consumer price index [16]. The producer price index and the producer price index for services use Classification of Products by Activity (CPA) classifications [17], which are in accordance with NACE classifications and comprise all goods and services. The consumer price index is used for construction, wholesale, and retail trade industries, with some exceptions; for more details on index choices, see Sheet 4.

Calculations of the adjusted turnovers for 2019 and 2021 are based on annual changes, which are the changes of the relevant index relative to the corresponding time period a year previously [18]. Differences in the indices between 2020 and 2018, 2022, 2023, and 2024 are calculated using point figures, which express the price of the comparison period relative to the price of the base period [19]. If the statistics for different indices are partially incomplete (i.e. the annual change or point figure for certain years is missing), index values are taken from a higher level or closely related index. This is the case with 27 industries, as shown in Sheet 4.

Inflation adjustments for 2018 and 2019 are calculated by industry according to Eq. (5):

(5) $$T_{A,201x}=\frac{\left(\;\Delta I_{\left[201x-2020\right]}+100\right)\cdot T_{201x}}{100}$$

where *T*_A,201x_ is the adjusted turnover for 2018 or 2019; *∆I*_[201x–2020]_ is the change in index between 2018 and 2020 calculated using point figures or the annual change between 2019 and 2020; and *T*_201x_ is the turnover for 2018 or 2019.

Inflation adjustments for 2021, 2022, 2023, and 2024 are calculated by industry according to Eq. (6):

(6) $$T_{A,202x}=\frac{T_{202x}\;\cdot 100}{\Delta I_{\left[2020-202x\right]}+100}$$

where *T*_A,202x_ is the adjusted turnover for 2021, 2022, 2023, or 2024; *∆I*_[2020–202x]_ is the annual change between 2020 and 2021 or the change in index between 2020 and 2022, 2023, or 2024 calculated using point figures; and *T*_202x_ is the turnover for 2021, 2022, 2023, or 2024.

Inflation adjustments are not needed for the base year 2020. Reallocations between industries arising from the EPR reporting practices of parent and associated child companies in Jylhä et al. [1] are not performed here. To validate the approach, the data for 2020 are recalculated without reallocations to allow comparisons with the accounting used by Jylhä et al. [1] and to quantify differences between the reallocated and non-reallocated calculations.

## Limitations

Company- and industry-specific raw data similar to that accessed by Jylhä et al. [1] are not available for 2018–2019 and 2021–2024. The extrapolation of the sample-based accounts to different years does not consider the transfer of companies between industries; this is necessary to allow compilation of the initial packaging accounts. It is also assumed that packaging intensities remain constant over time, so the results may not be reliable over longer periods as different industries change their packaging usage.

## Ethics Statement

The authors have read and follow the ethical requirements for publication in Data in Brief and confirm that the current work does not involve human subjects, animal experiments, or any data collected from social media platforms.

## CRediT authorship contribution statement

**Henna Jylhä:** Methodology, Formal analysis, Visualization, Writing – original draft, Writing – review & editing. **Annika Johansson:** Methodology, Visualization, Writing – original draft, Writing – review & editing. **Jani Salminen:** Methodology, Writing – original draft, Writing – review & editing, Funding acquisition, Supervision, Data curation.

## Data Availability

ZenodoA dataset for material-specific packaging volumes in Finland, 2018–2024 (Original data)
